# Impact of meal frequency on gastroesophageal reflux disease following laparoscopic sleeve gastrectomy

**DOI:** 10.3389/fsurg.2026.1768329

**Published:** 2026-06-16

**Authors:** Abdullah Almunifi, Muqrin A. Almuqrin, Ali Q. Al Qahtani, Abdullah Albarrak, Shiela Sadili-Fajardo, Abdulaziz A. AlMulhem, Eyad Wohaibi, Mohammad A. Al Mofarreh

**Affiliations:** 1 Department of Surgery, College of Medicine, Majmaah University, Al-Majmaah, Saudi Arabia; 2 Department of Mathematics, College of Sciences, Majmaah University, Al-Majmaah, Saudi Arabia; 3 Department of Gastroenterology, Al-Mofarreh Polyclinic, Riyadh, Saudi Arabia; 4 Department of Surgery, College of Medicine, Shaqra University, Shaqra, Saudi Arabia; 5 Department of Surgery, King Faisal Specialist Hospital and Research Center, Riyadh, Saudi Arabia

**Keywords:** bariatric surgery, gastroesophageal reflux disease, laparoscopic sleeve gastrectomy, meal frequency, Saudi arabia

## Abstract

**Background:**

Saudi Arabia has a high obesity rate (> 35%), increasing the risk of gastroesophageal reflux disease (GERD) and presents major health risks. Laparoscopic sleeve gastrectomy (LSG) is an effective bariatric surgery that reduces mortality. Controversies exist regarding the recommended meal frequency following bariatric surgery, as some patients experience increased GERD symptoms postoperatively. In this study, we describe the association between meal frequency and GERD at least one year after LSG.

**Methods:**

We conducted a retrospective cohort study based on data collected from electronic medical records over a six-year period (2016–2022) at a private gastroenterology clinic in the Kingdom of Saudi Arabia. Clinical, endoscopic, and radiological examinations of the upper gastrointestinal tract were reviewed as indicated. Paired-sample t-tests and two-way analyses of variance (ANOVA) were performed.

**Results:**

The study included 109 participants (62 women, 47 men) aged 17–66 years, mostly Saudi Arabian. All patients lost weight, with Body mass index (BMI) decreasing from 46.37 to 31.24 kg/m^2^. Primary complaints were heartburn (77.1%), epigastric pain (70.6%), retrosternal burning (50.5%), nausea (35.8%), and vomiting (30.3%). Only 15.6% had a history of preoperative endoscopy. Typical long-term daily postoperative meal patterns were 1–2 meals (43%), 3–4 meals (28.4%), 5–6 meals (8.2%), and more than six meals (4.6%). Most patients with RO consumed 1–2 meals (19.2%) or 3–4 meals (15.6%), compared to 5–6 meals (4.6%) and over six meals (0.9%). No significant association was observed between meal frequency and RO (*p* = 0.09, 0.24, 1.00).

**Conclusions:**

Large daily meals, with few frequent meals that meet daily caloric requirements, did not show statistically significant association with reflux oesophagitis (RO).

## Introduction

According to the World Health Organization (WHO) classification, overweight is defined as a body mass index (BMI) of 25.0–29.9 kg/m², obesity as a BMI of 30.0 kg/m² or above (with class I obesity defined as 30.0–34.9 kg/m², class II as 35.0–39.9 kg/m², and class III or severe obesity as 40.0 kg/m² or above) (1). In 2014, obesity affected more than 600 million people worldwide, and since 1980, its incidence has doubled (2). By 2030, 38% of people worldwide will be overweight, and another 20% will be obese (3). If the current trend continues, 15% of children and adolescents and 40% of the United States population will be obese by 2025 (4, 5). The obesity rate in the Kingdom of Saudi Arabia (KSA) is one of the highest in the world, with over 35% of the population being overweight. This percentage increased by an average of 0.64% annually between 2012 and 2016 (6–8). Global studies have contributed to the understanding of obesity as a complex, multifaceted disease that affects many aspects of physical and psychological health and is both treatable and preventable (9, 10). Up to 50% of people who are severely obese have gastroesophageal reflux disease (GERD), which is linked to obesity (11).

According to WHO data, over 1.9 billion individuals worldwide were overweight in 2016, with over 650 million classified as obese, a trend that has spread to low- and middle-income countries including Saudi Arabia (12, 13). Obesity is strongly associated with chronic conditions such as type 2 diabetes, cardiovascular disease, and certain cancers (14–19).

According to some researchers, LSG and GERD symptoms present before surgery are connected. Others have discovered that patients who undergo LSG may develop GERD or erosive esophagitis for the first time and that these patients frequently notice a considerable worsening of their symptoms (20–24). Some dietary guidelines recommend consuming 4–6 smaller meals (25, 26), while others advise consuming only 2–3 daily (27, 28). Some data exist concerning postoperative eating disorders, as these may correlate with poor postoperative results, which may lead to nutritional deficiencies (29–31).

GERD presents itself as heartburn and regurgitation. GERD can interfere with an individual's daily life. GERD can also cause issues such as erosive esophagitis. GERD is common among people with severe obesity (33). Some statistics indicate that half of all people with severe obesity suffer from GERD. As the popularity of bariatric surgery has grown to combat severe obesity, it has become important to understand how it influences GERD (34–36). The most common type of bariatric surgery is laparoscopic sleeve gastrectomy. LSG offers excellent long-term weight loss. LSG also has the potential to reduce mortality risk in individuals with severe obesity (11).

LSG has also been recommended as the first surgical option to combat metabolic disorders due to severe obesity. However, LSG's relationship with GERD is complicated. Some individuals with GERD experience improvement with LSG (37). However, most research has shown that individuals with GERD experience worsening or onset of GERD post-LSG (38). The symptoms are not consistent with what doctors observe. The post-surgery diet has an effect on GERD. The post-surgery diet is an important variable we can alter. Some research recommends eating 4 to 6 small meals daily. Other research recommends eating 2 to 3 large meals daily (39).

Although the frequency of meals has an effect on an individual's diet post-LSG, few research studies on post-LSG diet patterns exist (40). Only a few research studies on post-LSG diet patterns have examined how often an individual eats post-LSG (34–37). Since post-LSG diet patterns can alter stomach capacity and stomach pressure, which are also connected to GERD, it has become important to understand how post-LSG meal frequency patterns are connected to GERD (41).

Aside from symptoms, GERD can manifest as “reflex esophagitis” or “RE,” an endoscopic manifestation that reflects the presence of stomach acid on the esophageal lining. This is important because it is recognized that “symptoms and mucosal illness can be mismatched, with large symptoms not associated with RE, or with RE in association with mild symptoms” (24, 40). In severe obesity, GERD is common, with reports indicating that “up to half of very obese individuals have symptoms or disease of reflux” (11). With bariatric surgery increasingly being performed to treat severe obesity, it is necessary to understand how bariatric surgery affects both GERD symptoms and RE (35).

Therefore, this study aimed to examine, using routinely recorded clinical and diet-history data from a post-LSG cohort, the relationship between the number of meals consumed per day and the presence of GERD-related symptoms and reflux oesophagitis on upper GI endoscopy, particularly in the long-term period (>1 year) after surgery.

## Materials and methods

We examined existing data from Al-Mofarrah Polyclinic, a private gastroenterology clinic in Saudi Arabia that provides care to patients from all over the country. We analyzed data from January 2016 to December 2022 to determine whether the frequency of daily meal consumption was associated with gastroesophageal reflux disease (GERD) and reflux esophagitis (RO) following laparoscopic sleeve gastrectomy (LSG).

All data were extracted from the electronic medical records. No new interviews or surveys were conducted. Demographics, weight, and BMI, as well as clinical, lab, imaging, endoscopy, and pathology information, were extracted. Meal frequency was not determined from new memory recall. It was already documented in the system, mostly as follow-up information from dietitians and standard dietary histories during follow-up visits after LSG, so we used that information to determine exposure. If there were multiple entries, we used the entry closest to the time of the outcome.

Ninety-nine patients fulfilled the criteria and had enough information to determine both meal frequency and reflux outcomes. Although we did not perform *a priori* sample size or power calculation, we acknowledge that this is a limitation of our study. A *post hoc* power analysis indicated that with 109 participants, the study had limited statistical power to detect small effect sizes, which may have contributed to the non-significant findings. The sample was determined by the number of post-LSG patients available in the database during the study period who had complete information on the key variables. This method may not be sensitive to very small differences, but it allows us to try to find patterns and ideas for future studies that are specifically designed to find them. We acknowledge that our analytical approach, in which participants were grouped by the presence or absence of reflux oesophagitis (RO) rather than by meal frequency as the exposure variable, is more consistent with a comparative or case-control framework than a classical retrospective cohort design. Ideally, a cohort analysis would stratify participants by meal frequency and calculate the relative risk of developing RO across exposure groups. We recommend that future studies adopt such an approach with appropriate statistical consultation to strengthen causal inference.

Patients could be included if they underwent LSG during the study period, if they fulfilled bariatric surgery criteria (BMI >40 kg/m², or BMI >35 kg/m² with obesity-related health issues), and if data for meal frequency and reflux were available. We excluded patients with unknown meal frequency, missing key outcome data, or missing endoscopy data if endoscopy-based grouping was required. We also excluded patients who had other surgeries than LSG or other conditions mentioned in the records that might cause confusion in symptom analysis.

Outcomes for GERD/RO were obtained from the records and, if available, objective data. Symptoms such as heartburn, regurgitation, difficulty swallowing, and nausea were obtained from the records. RO was present if reflux esophagitis and associated findings (including hiatal hernia, if mentioned) were observed during upper GI endoscopy. Not all patients had endoscopy post-LSG; only those in the final group had endoscopy and sufficient data for analysis. All endoscopies were performed using a Fujinon EG-590WR video scope. Biopsies of the esophagus, stomach body, antrum, and duodenum were obtained as indicated, and pathology results were reviewed when available.

By duration, “long-term” refers to measurements obtained more than one year post-surgery. “Portion size” refers to the size recorded in the records—usually a small meal that complies with post-bariatric nutritional guidelines and does not exceed the patient's daily calorie intake.

IBM SPSS Statistics version 25 was used for statistical analysis. Continuous data were described using relevant descriptive statistics, and intergroup comparisons were made using tests such as paired *t*-tests and two-way ANOVA as appropriate. Significance was set at *p* < 0.05. The study was approved by the Institutional Review Board of King Fahad Medical City, Riyadh, Saudi Arabia (IRB00010471). Patient confidentiality was maintained, and data access was restricted to the researchers.

## Results

In total, 109 participants were included in this study. The majority of patients were aged 31–40 (35%) and female (57%) (Table 1). All patients lost weight, with their BMI decreasing from 46.37 kg/m^2^ to 31.24 kg/m^2^ *p* (0.000) (Table 2).

**Table 1:** Demographic characteristics and time since LSG among participants undergoing UGETE.

Age group (years)	*n*	Male, *n* (%)	Female, *n* (%)	Age range (years)	Mean age (years)	Post-op <1 year, *n* (%)	Post-op 1–4 years, *n* (%)	Post-op >4 years, *n* (%)
17–20	2	1 (50.0)	1 (50.0)	17–19	18.0	1 (50.0)	0 (0.0)	1 (50.0)
21–30	31	16 (51.6)	15 (48.4)	21–30	25.5	3 (9.7)	16 (51.6)	12 (38.7)
31–40	38	14 (36.8)	24 (63.2)	31–40	35.5	3 (7.9)	14 (36.8)	21 (55.3)
41–50	27	13 (48.1)	14 (51.9)	41–50	45.5	0 (0.0)	11 (40.7)	16 (59.3)
51–60	8	3 (37.5)	5 (62.5)	52–60	56.0	0 (0.0)	1 (12.5)	7 (87.5)
61–70	3	0 (0.0)	3 (100.0)	62–66	64.0	0 (0.0)	2 (66.7)	1 (33.3)
Total	109	47 (43.1)	62 (56.9)	17–66	40.75	7 (6.4)	44 (40.4)	58 (53.2)

BMI, body mass index; LSG, laparoscopic sleeve gastrectomy; UGETE, upper gastrointestinal endoscopy (as defined in Methods); Post-op, postoperative.

**Table 2:** Pre-operative and post-operative BMI by age group among participants undergoing UGETE after LSG.

Age group (years)	*n*	Pre-op BMI range	Pre-op BMI mean (SD)	Post-op BMI range	Post-op BMI mean (SD)
17–20	2	36.55–49.63	43.09 (3.52)	17.58–28.73	23.15 (4.27)
21–30	31	30.08–57.88	43.98 (—)	20.55–40.08	30.31 (—)
31–40	38	31.05–56.50	43.77 (—)	20.61–44.82	32.71 (—)
41–50	27	30.85–71.51	51.18 (—)	21.41–47.74	34.57 (—)
51–60	8	35.43–56.45	45.94 (—)	23.90–45.00	34.46 (—)
61–70	3	40.10–60.50	50.30 (—)	27.29–41.74	32.24 (—)
Total	109	30.08–71.51	46.37 (—)	17.58–47.74	31.24 (—)

BMI, body mass index; LSG, laparoscopic sleeve gastrectomy; Pre-op, preoperative; Post-op, postoperative; SD, standard deviation.

Only 17 patients (15.6%) had a history of preoperative endoscopy. All of these patients (100%) complained of epigastric pain, (88.2%) of heartburn pre-op, and (76.5%) of retrosternal burning (Table 3).

**Table 3:** Number of patients who underwent Pre-OP endoscopy: 17.

No.	Age range	Total	No.	Complaints					
				EP	HB	RB	Nau	V	Dph
1	17–20	2	0	1	1	0	0	0	0
2	21–30	31	3	4	4	4	1	1	0
3	31–40	38	7	6	5	4	3	3	0
4	41–50	27	5	3	3	4	1	1	0
5	51–60	8	0	1	1	1	0	0	0
6	61–70	3	2	2	1	0	0	1	1
TOTAL		109	17	17	15	13	7	6	1
			15.6%	100%	88.2%	76.5%	41%	35.3%	5.9%

EP, Epigastric Pain; HB, Heartburn; RB, Retrosternal burning; Nau, Nausea; V, Vomiting; Dph, Dysphagia; Ni, No informatio.

All 109 patients underwent endoscopic examination of their UGETEs 1 and 6 years after LSG. Their primary complaints were, in order, heartburn (77.1%), epigastric pain (70.6%), retrosternal burning (50.5%), nausea (35.8%), and vomiting (30.3%) (Table 4).

**Table 4:** Number of patients with obesity who underwent post-OP endoscopy after LSG (2016–2022): 109.

No.	Age in years	Total	Complaints						Endo Findings										
			EP	HB	RB	Nau	V	Dph	RO(LA)					G-itis	G-E	G-U	D-itis	D-E	D-U
									A	B	C	D	Total						
1	17–20	2	1	2	2	1	2	0	1	0	0	1	2	2	0	0	0	0	0
2	21–30	31	21	23	16	13	8	0	3	4	2	0	9	31	2	1	16	0	0
3	31–40	38	28	30	15	14	10	3	4	6	1	3	14	35	12	1	11	2	0
4	41–50	27	19	24	18	7	8	2	2	8	3	4	17	27	4	1	8	0	0
5	51–60	8	6	4	3	3	5	1	1	3	0	2	6	8	1	0	1	0	0
6	61–70	3	2	1	1	1	0	1	0	2	0	0	2	3	2	0	1	1	0
TOTAL	109	77	84	55	39	33	7	11	23	6	10	50	106	21	3	37	3	0	
		70.6%	77.1%	50.5%	35.8%	30.3%	6.4%	10.1%	21.1%	5.5%	9.2%	45.9%	97.2%	19.2%	2.8%	33.9%	2.8%	0%	

EP, Epigastric Pain; HB, Heartburn; RB, Retrosternal burning; Nau, Nausea; V, Vomiting; Dph, Dysphagia; RO, Reflux oesophagitis; LA, Los Angeles Classification of Reflux Oesophagitis, G-itis; Gastritis, G-E, Gastric erosion; G-U, Gastric ulcer; D-itis, Duodenitis; D-E, Duodenal erosion; D-U, Duodenal ulcer.

Table 5 shows that 44 (40.3%) of all post-LSG patients developed RO. Among these patients, those consuming 1–2 and 3–4 daily meals had RO rates of 19.3% and 15.6%, respectively, compared to 4.6% and 0.9% for those who ate 5–6 and more than six meals per day. However, the majority of patients who developed RO (44 in total) were consuming 1–2 daily meals (47.7%) and 3–4 meals (38.6%), compared to 11.4% and 2.3% for those consuming 5–6 and more than six daily meals, respectively.

**Table 5:** Reflux oesophagitis post-LSG according to meal frequency and time differences.

No	Years post-LSG	Post-op meals/die																*Grand Total*		*P*-value
		*Without RO/NOM*								*RO/NOM*										
		1–2	3–4	5–6	*>6*	*ni*	*Total*	*%*	*All %*	1–2	3–4	5–6	*>6*	*Total*	*%*	*All %*	*NMi*	*No.*	*All %*	
1	<1 year	3	2	0	0	1	6	10.2	5.5	1	0	0	0	1	2.3%	0.9%	0	*7*	6.48%	0.09
2	1–4 years	10	7	4	1	2	24	40.7	22	10	7	1	0	18	40.9%	16.5%	2	44	40.4%	0.24
3	>4 years	13	6	2	1	7	29	49.1	26.6	10	10	4	1	25	56.8%	22.9%	4	58	53.2%	1.00
Total		26	15	6	2	10	59			21	17	5	1	44			6	109	100%	
%		44.1%	25.4%	10.2%	3.4%	16.9%	100%			47.7%	38.6%	11.4%	2.3%	100%						
ALL %		23.8%	13.8%	5.5%	1.8%	9.2%	54.1%			19.3%	15.6%	4.6%	0.9%	40.3%			5.5%	100%		

N, Normal; Ni, No information; RO, Reflux oesophagitis; LA, Los Angeles Classification of Reflux Oesophagitis; NMi, No meal info; NOM, Number of meals; OP, operation.

Furthermore, RO rates increased to 0.9%, 16.5%, and 22.9% for all patients, compared to 2.3% (*P* = 0.09), 40.9% (*P* = 0.24), and 56.8% (*P* = 1.00) for those who developed RO within less than one year, 1–4 years, and more than 4 years postoperatively, respectively.

## Discussion

In this study, we aimed to evaluate the association between postoperative meal frequency and GERD, specifically RO, in patients following LSG. In a six-year retrospective analysis of 109 patients, significant weight loss was observed alongside common gastrointestinal symptoms such as heartburn, epigastric pain, and retrosternal burning. Our main finding is that patients consuming fewer, larger meals are more likely to experience RO at least one year after LSG. These results suggest that a dietary pattern characterized by a limited number of large meals, which fully meet daily caloric requirements, may lead to excessive filling of the gastric sleeve, thereby increasing the risk of GERD.

GERD is common among individuals with severe obesity, with up to 50% affected (42, 43). Following LSG, 25% to 40% of patients may develop *de novo* GERD and approximately 30% show erosive esophagitis (16, 45, 46). Although weight loss and decreased intra-abdominal pressure following LSG may relieve existing GERD, many patients develop new or worsening reflux symptoms (42). Our cohort demonstrated a similar pattern, with 45.9% of patients developing RO on post-operative endoscopy.

LSG is known to alter dietary intake and food preferences (31, 39), and postoperative eating disorders may be associated with poor surgical outcomes and nutritional deficits (36). These dietary changes following LSG are relevant to our findings, as they may influence both meal frequency patterns and the development of reflux symptoms.

Dietary guidelines following bariatric surgery vary, with some centers recommending 4–6 smaller meals daily and others advising 2–3 larger meals (25–28). Because LSG permanently reduces stomach capacity, portion size is an important consideration; overfilling the reduced stomach may increase intragastric pressure and expedite gastric emptying, potentially contributing to GERD and RO (47). Our data are consistent with this hypothesis, as we observed that RO was more prevalent among patients consuming fewer, larger meals.

We focused on the post-LSG meal frequency (meal numbers) rather than diet or food habits. Many studies and nutritional guidelines from different centers have examined food intake, eating behavior, and dietary intake after LSG (25–31). Despite our best efforts, we found only three global studies on patients with LSG that reported on daily meal frequency or daily meal numbers. Vakhshoori et al. observed that women who ate 6–7 or > eight snacks and meals daily had 38% and 43% lower incidences of GERD than those who ate less than three snacks or meals (32). Laurensius et al. averaged 5.4 meals per day (33). According to Lye et al. (34), early postoperative overeating increases the risk of reflux and intragastric pressure, according to Lye et al. (34). However, he did not elaborate on meal frequency.

Our Data showed that the RO was much greater when eating a few larger meals and less when eating frequent small meals. Furthermore, it significantly increased with postoperative time.

We emphasize that active patient education regarding appropriate portion sizes, calorie intake, and the importance of endoscopic surveillance after LSG should be integral to postoperative care (8, 44).

This study had several limitations. First, missing data, such as unrecorded calorie counts, could have biased our results. Second, although we described this as a retrospective cohort study, we grouped participants by the presence or absence of RO (outcome) rather than by meal frequency (exposure), which is more consistent with a comparative or case-control design. A true cohort analysis would stratify by meal frequency and calculate the relative risk of developing RO; we encourage future investigators to adopt this approach with statistical consultation to strengthen causal inference. In addition, the retrospective design, single-center setting, and absence of a control group limit the generalizability of these findings. Prospective multicenter studies are needed to confirm these results and further clarify the relationship between meal frequency and post-LSG GERD symptoms.

Eating large meals throughout the day to satisfy calorie requirements may help contribute to the stretching of the stomach sleeve and reflux symptoms following laparoscopic sleeve gastrectomy. However, this study demonstrated that there was no significant relationship between the number of times patients eat meals and reflux esophagitis observed during endoscopy.

Therefore, although the distribution of calorie intake throughout the day may be physiologically beneficial, this study did not demonstrate a statistically significant relationship between meal frequency and reflux esophagitis. Future studies should investigate the relationship between meal patterns and reflux following LSG. Public health education remains vital in improving post-LSG dietary practices and expectations, but recommendations regarding meal frequency and reflux risk should be offered with caution until more definitive evidence emerges.

## Data Availability

The original contributions presented in the study are included in the article/Supplementary Material, further inquiries can be directed to the corresponding author/s.
